# The Sick and Wounded at Santiago

**Published:** 1898-08

**Authors:** 


**Monthly Review of Medicine and Surgery.**

*EDITORS:*

THOMAS LOTHROP, M. D. - - WM. WARREN POTTER, M. D

All communications, whether of a literary or business nature, should be addressed to the managing editor: 284 FRANKLIN STREET, BUFFALO, N. Y.

VOL. XXXVIII. AUGUST, 1898. NO. 1

### THE SICK AND WOUNDED AT SANTIAGO.

THE rapid development of war in eastern Cuba since our July edition went to press has tested the quality of both army and navy medical officers and the adequacy of their respective organisations to meet the requirements of actual battle.

From the sailing of General Shafter's fifth army corps, June 14th to the capitulation of Santiago, July 16, 1898—only a little more than a month—was a period full of momentous events. The landing of the army through the surf without adequate lighters, chiefly through the agency of small boats, with the loss of but two lives, was a remarkable achievement; but it presaged the victorious conquest that was soon to follow. From the preliminary skirmish of Wood and Roosevelt's rough riders at La Quasina, June 24th, to the sanguinary conflicts at San Juan and El Caney, July 1st and those of the still further contracted lines around the doomed city on the 2d, presents a picture of sturdy valor, and enduring courage that has arrested the attention of the civilised world.

The price of the victories on those fields, according to General Shafter's official statement in General Order No. 26, is a total of 1,598 officers and men distributed as follows: killed, 230; wounded, 1,284; missing, about 84. It has lately been learned that the Spanish loss was 400 killed and more than 2,000 wounded. While the conditions for the care of our wounded were not the most favorable, owing to the bad state of the roads and other circumstances incident to the rapidity of movement, yet it has been officially learned that the disabled soldiers were adequately treated within a reasonably short time and with little if any more confusion than usually results from great battles.

The character of the wounds, too, caused by the Mauser rifle was less severe than at first feared. The brass jackets of the missiles have in some instances caused considerable laceration, but there have been fewer amputations than usual in proportion to the total number wounded. Flesh wounds, as a rule, have healed promptly and many soldiers who received slight wounds are already on duty again in the ranks. In support of the general statements contained in the foregoing paragraphs we quote the remarks of Lieutenant-colonel Charles Smart, deputy surgeon-general United States Army, who, in reply to a question as to the nature of wounds made by the Mauser bullet, is reported to have replied substantially as follows :

After the fight at La Quasina and the battles of July 1st, 2d and 3d, a report became current that the Spaniards were firing explosive bullets, which spread in such a way as to cause great laceration. That was the first report and it has been repeated frequently ever since.

The fact is that the Mauser bullets used by the Spaniards in the battles we have had in Cuba make what surgeons call a "humane wound." They drop the man at the time he is struck and take him from the firing line, but if they do not kill him then and there he gets well.

Of all the men wounded in General Shafter's command I have heard of only one case of an amputation of a limb as the result of a bullet wound, where the bone has been shattered. The Mauser bullet does not leave a wound nearly so difficult to heal as do the bullets used in the Springfield rifle.

During the civil war, when a man was shot through the lungs by a bullet from a Springfield rifle he was almost sure to die in a few days, or in a few months from consumption or pneumonia or some other affection brought on by the wound. The Mauser bullet will pass through the lungs and the patient will recover. It does not crush bones so that amputations are necessary. A wound made by such a bullet, if it does not affect a vital part, heals as readily as would a slight cut.

When the medical history of this war is written it will show the most remarkable results ever achieved in antiseptic treatment of wounds. Every regiment except one under General Shafter, was provided with a small package marked "First aid to the wounded." This package was carried in the hip pocket by all the men.

This was extremely fortunate, because after the transports discharged their passengers they sailed away somewhere with the medicine chests on them. When a soldier was wounded the doctor reached for the "first aid" package, carried by the soldier himself, and, applying it to the wound, completed the bandaging then and there. This antiseptic treatment caused the wound to heal without the formation of pus and has shown wonderful results.

The sick and wounded of the navy, recently brought north by the Solace, the wounded being from the conflict with Admiral Cervera's squadron off Santiago, July 3d, twenty-two in number, are reported by Surgeon-general Van Reypen, who recently inspected the Solace, to be in excellent condition. These were chiefly shell wounds and those produced by splinters.

While it is true that yellow fever has appeared, it is yet equally true that it is mild in form and not at present epidemic in character. A few deaths have occurred, but most of those seized with the disease are recovering. Camps have been changed to healthful locations whenever practicable and the malady appears to be well in hand.

There is no good reason why this infection should not be controlled as well as other forms that have fallen under the bans of science. Spain has been *particips criminis* to its propagation for centuries, but, happily, her rule is at an end, or nearly so, in this hemisphere. If there were no other good to grow out of the war this one would adequately justify it.

The latest accounts encourage the belief that the timely action of the surgeon-generals of the army and navy, taken at the outset for the control of yellow fever, are yielding results that are most gratifying and that not long hence the disease will be stamped out.

The situation at Santiago, therefore, from a medical and surgical viewpoint, is such as to bring credit to the medical service of both army and navy.

